# Beyond Their HIV Status: the Occurrence of Multiple Health Risk Behavior Among Adolescents from a Rural Setting of Sub-Saharan Africa

**DOI:** 10.1007/s12529-020-09877-6

**Published:** 2020-03-25

**Authors:** Derrick Ssewanyana, Charles R. Newton, Anneloes van Baar, Amin S. Hassan, Alan Stein, H. Gerry Taylor, Fons Van De Vijver, Gaia Scerif, Amina Abubakar

**Affiliations:** 1 Centre for Geographic Medicine Research Coast, Kenya Medical Research Institute (KEMRI), P. O Box 230, Kilifi, 80108 Kenya; 2 Utrecht Centre for Child and Adolescent Studies, Utrecht University, Utrecht, The Netherlands; 3 Department of Psychiatry, University of Oxford, Oxford, UK; 4 Department of Pediatrics, Center for Biobehavioral Health, Nationwide Children’s Hospital Research Institute, The Ohio State University, Columbus, OH USA; 5 Department of Culture Studies, Tilburg University, Tilburg, The Netherlands; 6 Department of Experimental Psychology, University of Oxford, Oxford, UK; 7 Department of Public Health, Pwani University, Kilifi, Kenya; 8 Institute for Human Development, Aga Khan University, Nairobi, Kenya

**Keywords:** Health risk behavior, HIV, Adolescents, Sub-Saharan Africa, Latent class analysis

## Abstract

**Background:**

Health risk behaviors during adolescence may cluster into patterns that might be predicted by specific factors, among which HIV may have an important role.

**Method:**

In a cross-sectional study conducted between 2017 and 2018, clustering of HRB and its associated factors was investigated in rural Kenya among 588 adolescents (36% perinatally HIV infected; 28% perinatally HIV exposed but uninfected; and 36% HIV unexposed/uninfected). Latent class analysis of 22 behaviors followed by multinomial logistic regression were conducted. Four risk behavior classes were identified.

**Results:**

No significant differences were found in behavioral class membership across the three HIV groups (*p* = 0.366). The risk of membership to the higher risk behavioral classes relative to class 1 (the substance and drug abstinent low risk takers) increased with older adolescent age (*p* = 0.047), increased among adolescent who experienced mental distress (*p* < 0.001), and those who felt unsafe in their neighborhood (*p* < 0.002). Better working memory (*p* = 0.0037) was found to be protective.

**Conclusion:**

The results highlight a need to include screening and interventions for internalizing mental health problems and deficits in executive functioning, as well as steps to involve family members and communities to address psychosocial risk factors in adolescents in Kenya.

## Introduction

The emergence of a sub-population of adolescents living with HIV, especially in Sub-Saharan Africa (SSA) [1, 2], has been met with growing research interest on health risk behavior (HRB) of this group [3, 4]. HRB is often initiated or consolidated during adolescence [5] and heightens vulnerability to sickness and mortality by compromising development and successful achievement of adolescents’ expected social roles [6]. Sub-optimal HIV care and treatment outcomes like poor antiretroviral therapy (ART) adherence [7], sexually transmitted co-infections [8], and virologic failure [9] have also been linked to HRB. Most studies on HRB among adolescents living with HIV in SSA report a high prevalence (20–60%) of risky sexual behavior and sexual victimization as well as substance use behavior, especially among males in late adolescence [3, 4]. These findings make HRB an urgent target for intervention among adolescents living with HIV.

Amidst the growing body of evidence on HRB of adolescents living with HIV in the SSA, most studies only report on a few forms of HRB and usually in an isolated manner [3, 4]. However, reporting of HRB in an isolated manner is not comprehensive as cross-cultural findings indicate a common tendency for multiple forms of HRB to co-occur as risk behavior clusters or bundles during adolescence [10–13]. The mechanisms explaining HRB clustering are still poorly understood and thus further research on this topic is needed [10]. Clustering refers to an observed proportion of a combination of risk factors that exceeds expectations [14]. Notably, HRB clustering or co-occurrence underlies the concept of unhealthy lifestyle [6], which also increases risk for future chronic conditions [15] and can complicate HIV treatment and care outcomes [16]. To the best of our knowledge, none of the published studies from SSA has examined HRB clustering and its underlying factors among adolescents living with HIV. There have also been observations that HRB clustering is most prevalent among disadvantaged sections of the population and that this is partly attributable to specific shared risk factors, such as social determinants of health [2, 10]. Identifying clusters of behavior and their predictors within a sub-population can help to identify the most vulnerable adolescents and facilitate the design of more comprehensive health promotion planning and intervention programs [10]. Besides the fragmented reporting on HRB, many past studies on HRB among adolescents living with HIV in SSA lack details on response rates. Some do not clarify the route of HIV infection (i.e., *vertical* transmission from an HIV positive woman to her child during pregnancy, child birth or breast feeding, or *horizontal* transmission through contact with infected body fluids during sex or through using shared objects). Other studies do not clarify their participants’ awareness of their HIV status, and a number of studies lack appropriate comparison groups [3, 4]. Studies that compare the occurrence of multiple forms of HRB in perinatally HIV-infected adolescents, to that among perinatally HIV exposed but uninfected adolescents in SSA, are inexistent.

Noteworthy, most adolescents born to HIV-infected mothers in SSA dwell in vulnerable backgrounds characterized by several familial, environmental, and biomedical factors which may adversely impact their behavioral health [2, 17, 18]. We hypothesize that perinatal HIV infection and exposure is associated with HRB clustering. In addition, this association could largely be explained by a combination of common risk factors experienced during adolescence and additional unique factors, resulting from perinatal HIV infection and exposure. These two sets of underlying factors for HRB are however complex to disentangle [19]. It is plausible that the additional risk from HIV-specific factors results in a heightened level of vulnerability. Therefore, a greater burden of HRB clustering among adolescents with perinatal HIV infection and exposure is expected, compared to their unaffected adolescent peers. Indeed, research from SSA settings like Kenya [17, 20] and South Africa [18] indicates a disproportionately greater psychosocial disadvantage among perinatally HIV-infected adolescents than the uninfected peers from similar settings.

Mental distress and other mental illness resulting from psychosocial adversity is one of the unique factors attributable to perinatal HIV infection, which may potentially heighten the risk for HRB clustering in this sub-population. Indeed, psychiatric comorbid conditions occur at high rates in people living with HIV [21]. For perinatally HIV-infected adolescents, sources of mental distress/illness often arise from recurrent and cumulative psychological stressors of coping with HIV infection, traumatic and adverse childhood experiences (e.g., loss of close family members), responsibility of taking care of siblings and ill family members, violence, stigma and discrimination, absenteeism from school, and inconsistent guardianship among others [2, 18, 22, 23]. Poverty within households affected by HIV further complicates adversity of the household members [24]. Research from SSA confirms that childhood adversity heightens the risk of HRB such as risky sexual behavior and substance use among perinatally HIV-infected adolescents and youth [18]. Noteworthy, depression and other forms of mental illness like anxiety are also reported as strongly and independently associated with HRB in adolescence [25, 26].

Furthermore, a higher burden of poor neurocognitive functioning among perinatally HIV-infected children and adolescents (including those on HIV treatment) than that of their uninfected peers has been consistently reported in studies in SSA [27, 28] and other parts of the world [29, 30]. HIV-associated neurocognitive dysfunction has been linked to adverse effects of HIV infection [31, 32] and toxicity from prolonged exposure to HIV medication (in utero, through breast milk or treatment) [33], which may cause neuropathy and thus impairment within the frontal and pre-frontal regions of the brain. Neuropathy within the prefrontal cortex regions hampers important brain maturation processes and optimal development of subtle neurocognitive abilities like executive functions (EF) [34]. Executive functions are shown to play a crucial role in behavioral regulation during adolescence [5]. Correspondingly, research findings indicate the presence of significant weaknesses in EF domains, such as working memory, planning, and inhibitory control among adolescents who engage in substance use, violence, and sexual risk behavior [35–37]. EF’s significance in adolescents’ risk taking has also been linked to the dual brain system of adolescence [5], characterized by a temporal lapse between onset of the socio-emotional system at puberty (which increases reward-seeking tendencies) and the slower maturation of the cognitive control system (which improves individuals’ capacity for self-regulation) during later adolescence. This temporal lapse explains why risk taking propensity tends to heighten at the onset of puberty and subsequently decline in older adolescence stage [5]. Moreover, EF deficits in planning, inhibition, working memory, cognitive flexibility, problem solving, and processing speed are increasingly reported among youth with perinatal HIV infection [30]. Other factors linked to EF outcomes include socio-economic status [38], nutritional status [39], and educational achievement [40]. We therefore hypothesize that EF deficits play an important role in predisposing perinatally HIV-infected adolescents to a high burden of HRB clustering. This stated, none of the studies on HRB of adolescents living with HIV in SSA has investigated the potential impact of EF on HRB.

Some of the commonly documented risk factors for HRB in the general adolescent population, which may also play a role in HRB clustering, include coping challenges arising from rapid cognitive, physical, social, emotional, and sexual developmental changes [5, 41]; individual level factors like religiosity, age, sex, self-esteem, and risk perception [42–44]; family level factors like substance use by close family members, household poverty, parent-adolescent connectedness, and family history of mental illness [42, 45, 46]; school environment factors [42, 47]; peer influence [43]; and neighborhood characteristics and broader/macro level factors like policy and legal framework [48, 49].

Kenya is one of the countries in SSA with a large and youthful (15–24 years) HIV sub-population that comprises 12% of the total people living with HIV [50]. Nevertheless, studies on HRB of adolescents living with HIV in Kenya barely exist. In rural settings such as coastal Kenya, a high psychosocial burden coupled with suboptimal HIV treatment and lack of health and social services exacerbate problems, faced by adolescents living with HIV [17, 51, 52]. Within such settings, adolescents living with HIV may experience high vulnerability to HRB, which may adversely impact their HIV treatment outcomes and general health. Despite these circumstances, there is still a dearth of evidence on the lifestyle of adolescents living with HIV, as well as on the general adolescent sub-population from rural Kenya. In order to reduce the existing knowledge gaps, HRB clustering was investigated in rural coastal Kenya among perinatally HIV infected, perinatally HIV exposed but uninfected, and HIV unexposed and uninfected adolescents. We explored the extent to which perinatal HIV infection influences HRB clustering during adolescence, while controlling for relevant confounding factors tested in a directed acyclic graph (DAG).

## Methods

### Participants and Procedures

The first wave of data collection was conducted between November 2017 and October 2018, providing a baseline assessment for the ongoing longitudinal study, the Adolescent Health Outcomes Study (AHOS). The study was conducted at the Centre for Geographic Medicine Research-Coast at the Kenya Medical Research Institute (CGMRC-KEMRI) and all the participants were residents of Kilifi County at the coast of Kenya. About 1.4 million people resided in Kilifi County by 2016 of whom the majority (61%) were rural dwellers and 22% were aged 10–20 years [53]. Kilifi is termed as a “medium HIV county” with 45 per 1000 affected of whom 6000 (19%) were youth and young adults, aged 15–24 years [50]. About 891 km^2^ of Kilifi County is covered by Kilifi Health and Demographic Surveillance System (KHDSS) [54], a region with various ongoing CGMRC-KEMRI research activities.

Perinatally HIV infected and perinatally HIV exposed but uninfected adolescents and their caregivers were recruited through sequential sampling from all families that attended HIV clinic days at eight HIV treatment and care clinics at health facilities (hospitals and health centres) in Kilifi County. Recruitment was conducted by a trained research assistant in liaison with health workers at the participating HIV treatment facilities. Some of the perinatally HIV exposed but uninfected adolescents and their caregivers were also recruited by visiting families affected with HIV within their community with the assistance of a community health worker based at an HIV clinic. HIV unexposed and uninfected adolescents were randomly sampled among households within the KHDSS using the KHDSS population register [54].

Medical records at the health facilities were used to confirm perinatally HIV-infected adolescents’ HIV status. As part of the eligibility criteria, the adolescents had to be fully aware of their HIV status and that of their biological mother. The adolescent’s awareness of maternal HIV status was an important criterion for abating the ethical dilemma and emotional burden for example, tension and mistrust, that can potentially arise from the abrupt awareness of the source of HIV infection by the adolescents participating in this study and the members of their household. HIV exposure of perinatally HIV exposed but uninfected adolescents was ascertained from maternal medical records (antenatal care cards) confirming HIV infection of the mother during pregnancy. Additionally, recent medical records of the adolescent (if available) were used to ascertain that the perinatally HIV exposed but uninfected adolescent was HIV uninfected. The perinatally HIV exposed but uninfected adolescent also had to be aware of his or her biological mothers’ HIV status for study eligibility. HIV unexposed and uninfected adolescents were not directly tested for HIV but recruitment was restricted to those whose mothers willingly shared their HIV test results at the time of their pregnancy with the participating adolescent. For both perinatally HIV exposed but uninfected adolescents and HIV unexposed and uninfected adolescents, a brief screening checklist was utilized to exclude adolescents who had experienced severe childhood illness or were having recurring health problems so as to minimize the possibility of including HIV-infected adolescents among the control group. Besides, a more detailed assessment of the adolescents’ medical history, symptoms, and concerns was also done by a trained clinician during the assessments for data collection. All eligible adolescent participants had to be accompanied by a legal caretaker during their appointment for data collection at the CGMRC-KEMRI. Monetary reimbursement of 300 Kenyan shillings (about 3 US dollars) and a transport fee reimbursement were given to the accompanying caretaker and a snack was provided to all participants prior to the assessments. Ethical approval to conduct this study was obtained from the Kenya Medical Research Institute Scientific and Ethics Review Unit (KEMRI/SERU/CGMR-C/084/3454). Permission was also obtained from the Kilifi County government, department of health services (HP/KCHS/VOL.VIX/80).

The current study comprises 558 (199 HIV unexposed and uninfected, 158 perinatally HIV exposed but uninfected, and 201 perinatally HIV infected) adolescents aged 12–17 years. Initially, 638 potentially eligible adolescent participants (227 HIV unexposed and uninfected, 185 perinatally HIV exposed but uninfected, and 226 perinatally HIV infected) had shown interest in participating, but 560 ultimately took part. Of these 560, data on HRB outcomes from 2 participants was completely missing, therefore they were excluded. Non-response (*n* = 78) was mainly attributable to silent refusal (i.e., lack of follow-up in making or attending visits (*n* = 39, 50%), or to direct refusal of further contact with the research team (*n* = 9, 11.5%), to the failure to meet inclusion criteria (*n* = 7, 8.9%), and to participant relocation (*n* = 2, 2.6%)). Some eligible participants were not scheduled for an assessment after attaining the required sample size quotas (*n* = 21, 27%). Overall, the non-respondents did not differ by HIV group composition (34.6% HIV unexposed and uninfected, 34.6% perinatally HIV exposed but uninfected and 30.8% perinatally HIV infected) and sex distribution (*p* = 0.75). However, among the non-respondents, the HIV unexposed and uninfected group was the oldest (mean = 13.9 years, SD = 1.7, *p* = 0.002).

### Informed Consent

Informed consent was obtained from all the individual participants included in the study. Written parental or guardian consent as well as adolescents’ assent were obtained.

### Measures

#### Health Behavior Outcomes

HRB, the primary outcome of this study, was assessed using an audio-computer assisted self-interview (ACASI) of the Kilifi Health Risk Behavior Questionnaire (KRIBE-Q) in Swahili language. The KRIBE-Q was previously adapted and validated for use among the adolescent sub-population in Kilifi and is a reliable (Gwet AC1 = 0.82) measure for adolescents’ HRB [55]. Contextually relevant examples and explanations of HRB were utilized in the interviews for clarity. In summary, the reported behaviors comprised the following:

##### Injury or Violence-Related Behavior

Six injury and violence-related behaviors were reported: (i) was engaged in physical fights within the past 12 months; (ii) was seriously injured within the past 12 months; (iii) experienced dating violence (physical and/or sexual) within the past 12 months; (iv) was forced to have sexual intercourse; **(**v) was bullied within the past 12 months; and (vi) experienced suicidal behavior (ideation and/or attempt) within the past 12 months.

##### Sexual Risk Behavior

Three behaviors reported on sexual risk behavior were (i) early sexual debut (dichotomized initiation of sex before 14 years or at 14 years and above); (ii) engagement in transactional sex (victim and/or perpetrator) in the past 12 months; and (iii) condom nonuse during most recent sex.

##### Substance Use Behavior

Tobacco and alcohol use were categorized into one group as both are examples of licit substances that are largely accessible within the Kenyan context [56]. Marijuana and Khat were grouped together in a second category as central nervous stimulants [57], which was fairly accessible by youths within the study setting [58]. A third categorization was for other drug use. The five substance use behaviors reported were (i) lifetime use of tobacco or alcohol products, (ii) recent use (past 30 days) of tobacco or alcohol products, (iii) lifetime use of marijuana and Khat products, (iv) recent use (past 30 days) of marijuana and Khat products, and (v) lifetime use of other drugs.

##### Poor Hygiene Behavior

Two indications of poor oral hygiene and poor general body hygiene were reported. Respondents were asked how often they cleaned or brushed their teeth in a regular week and how often they washed their entire bodies with water and soap during a regular week.

##### Gambling

Gambling behaviors were captured by an item asking if the adolescent ever spent much more than they planned on gambling activities within the past 12 months.

##### Physical Activity and Sedentary Behavior

Three behaviors on physical activity and sedentary behavior were reported: (i) number of days of vigorous physical activity (at least 10 min at a time) during a regular week, (ii) number of days of moderate physical activity (at least 10 min at a time) during a regular week, and (iii) number of hours spent on sedentary activities during a regular day. Contextually relevant examples and explanations of sedentary behavior and vigorous and moderate forms of physical activity were utilized in the interviews for purposes of clarification.

##### Dietary Behavior

Dietary behaviors were captured by assessing the frequency of (i) fruit and vegetable consumption and (ii) fatty or fast food intake during a regular week.

#### Executive Functioning

Three core EF domains, namely working memory, inhibitory control, and cognitive flexibility [59], were assessed. All EF assessments were administered by a research assistant (trained in psychological and cognitive assessment) in quiet and properly lit rooms, arranged to minimize any form of distractions. Standardized procedures for administration of each EF test were followed.

##### Cognitive Flexibility

Five trails of the comprehensive trail making test (CTMT) were administered in numerical order following the standardized procedure [60]. Raw scores for each trial (number of seconds taken by an examinee to complete the trial) were recorded by the assessor. T-scores per trial were obtained from the CTMT examiner’s manual [60] and performance summarized by the *average T*-*scores* for all completed trials.

##### Inhibitory Control

The Stroop color and word test (SCWT) [61] was administered to assess inhibitory control. Study reports have shown a link between deficits in the ability to inhibit cognitive interference (inhibitory control) and impulsivity [62, 63]. From the three sections of the test (each timed for 45 s), the raw word score (words correctly identified), raw color score (correctly identified colors), and the raw color-word score (correctly identified color of ink for a contrasting name of color) were recorded by the assessor per examinee. The *interference* score was computed by subtracting the raw color score from the raw color-word score.

##### Working Memory

Participants were administered the backward digit span and the letter-number sequencing (LNS) subsets. These tasks have been previously modified for use with children, adolescents, and older populations within the study setting [64]. Scores were the total number of correct sequences (*total correct raw scores*) for both LNS and backward digit span tasks.

#### Other Variables

The adolescents’ age, sex, current educational level, and orphanhood status were captured and ascertained in the presence of their caretaker as well as from records such as birth certificates. The adolescents’ caretakers were also asked about their household socio-economic status using an assets index that has been extensively utilized in the Kilifi context [65]. Adolescents’ weight and height were measured according to recommended procedures [66]. Body mass index for age (BMI for age) and height for age were then computed using the WHO standards [67]. Items assessing parent-to-adolescent interaction, peer-to-peer relationship, and school attachment were from measures previously utilized in adolescent sub-populations [68–70]. The most suitable items for each of the three components were selected based on factor analytic approach. Additional items were taken from the KRIBE-Q [55] and assessed “household food insecurity” in the past 30 days (asking if one went hungry because there was not enough food at home), “feeling unsafe in their neighborhood,” “experience of mental distress” (feeling sad/hopeless almost every day for 2 weeks or more in a row) within the past 12 month, and “use of tobacco products by their caretaker/s.” A blood sample was taken from the perinatally HIV-infected adolescents and analyzed for CD4/CD8 cell count and HIV viral load concentrations.

### Statistical Analyses

All analyses were conducted in the STATA15 software package (StataCorp LLC). First, latent class analysis (LCA) [71] was performed to identify behavioral classes based on the 22 behaviors described above. Five models varying from one to five latent classes were generated and the Akaike information criterion (AIC) and Bayesian information criterion (BIC) were utilized to select the model with the best goodness of fit indices and lowest BIC values [72]. Assignment of participants to respective latent classes was based on their posterior probabilities of class membership. Entropy was also measured to indicate the level of separation between classes. Values of normalized entropy greater than 0.80 indicate that the latent classes are highly discriminating [73].

**Fig. 1:**
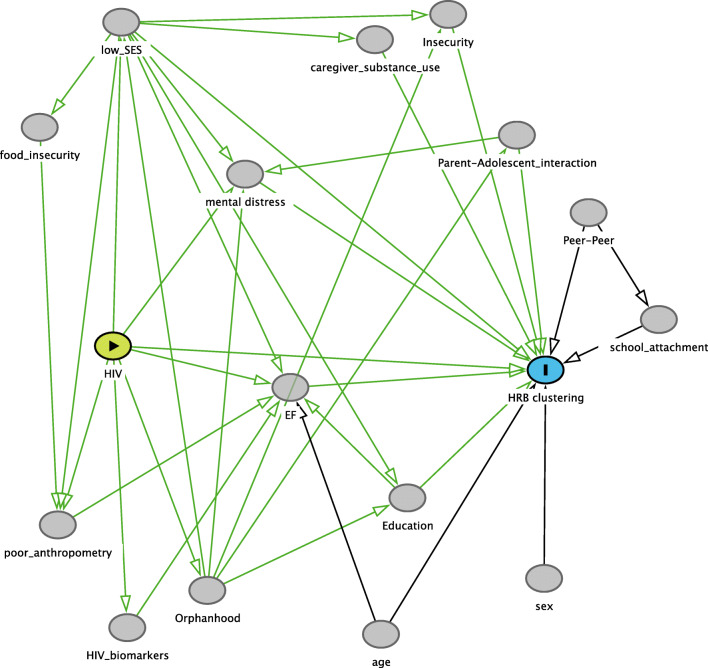
A directed acyclic graph (DAG) conceptualizing the effect of perinatal HIV infection on health risk behavior clustering among adolescents. HIV: perinatal HIV infection, HRB clustering: Health risk behavior clustering, EF: Measure of executive functioning domains of working memory, inhibitory control and cognitive flexibility, Low_SES: Low household socio-economic status, Sex: sex of the adolescent, Age: age of the adolescent, HIV_biomarkers: HIV treatment outcomes (CD4/CD8 cell count and HIV viral load concentrations), Poor_anthropometry: poor adolescent anthropometric measures of body mass index and height for age, Orphanhood: being an orphan, Education: adolescent’s current educational level, Food_insecurity: household food insecurity in the past 30 days, Mental_distress: Experience of mental distress within the past 12 months, Caregiver_substance use: Use of substances by the caretaker, Insecurity: Feeling unsafe in their neighborhood, Parent_adolescent interaction: Parent-to-adolescent interaction, Peer-peer: Peer-to-peer relationship, School_attachment: School attachment,  Exposure, : Outcome, : Other Variables, : Causal path, : Biasing path

The HRB composition, socio-demographic factors (sex, age, socio-economic status, education level, household food insecurity), biological factors (BMI, height for age, medical history, HIV status, CD4/CD8 cell count, HIV viral load concentrations), and psychosocial factors (mental distress, orphanhood, caregiver-adolescent interaction, caregiver tobacco use, school attachment, peer-peer relationship, feelings about neighborhood safety) of each class from the optimal model were summarized using descriptive statistics of proportions (%) and means. Bivariate analyses (Chi-square test or Fisher exact test for categorical variables and analysis of variance [ANOVA] with Bonferroni correction for post-hoc analysis for continuous variables) were used to test for significance of differences in HRB composition, socio-demographic, and biological and psychosocial factors across the behavioral classes. ANOVA with Bonferroni correction for post-hoc analysis was performed to identify differences in EF outcomes across classes. A directed acyclic graph (DAG) of the hypothesized exposure-outcome relationship was generated in DAGitty V2.3 open access software [74] (see Fig. 1). The exposure-outcome relationship relies on prior knowledge from other empirical studies and assumed contributory effects which were explained in the introduction section of this article. Using the DAG, we identified variables for adjustment (also known as minimum sufficient adjustment sets) in estimating the effect of perinatal HIV status on HRB clustering. DAGs are increasingly utilized in modern epidemiology and are found to be crucial in advancing investigation of causal relationships which involve multiple interrelated variables [75, 76]. DAGs are also useful for avoiding the introduction of collider bias (i.e., conditional associations introduced by selected covariates) and identifying confounding [77]. Multinomial logistic regression was conducted to investigate the association between perinatal HIV infection and HRB clustering while controlling for the variables identified from the DAG model as minimum sufficient adjustment sets. Multiple imputation was used for data missing at random on individual behavioral variables due to non-response [78].

## Results

### Sample Characteristics

The final sample comprised 558 adolescents (36% perinatally HIV infected, 28.3% perinatally HIV exposed but uninfected, and 35.7% HIV unexposed and uninfected) with a mean age of 13.7 (SD = 1.6). The perinatally HIV exposed but uninfected were slightly younger (13.3 (± 1.4) years) than the other groups, with the oldest being the perinatally HIV infected (14.1 (± 1.6) years) (*F* = 11.9, *p* < 0.001). Overall, 53% were females and no differences in sex were found across HIV groups (*p* = 0.72). Almost all participants (99%) attended school, with a majority (93%) being at primary education level. Group differences in education were not significant (*p* = 0.06). All the perinatally HIV-infected adolescents were enrolled on ART of whom 17.5% were prescribed protease inhibitor-based regimen (i.e., second line regimen).

### Behavioral Classes

The four-class solution was found to be the best fitting model as it had the lowest BIC (12,238.06) and adequate power for the entropy measure (0.85) to indicate a high level of discrimination by the 4 latent classes (see Table 1).

**Table 1:** Model fit information for the latent class models from 1 to 5 clusters (*n* = 558)

Number of clusters	Degrees of freedom	AIC	BIC	Entropy
1	24	12,628.51	12,732.3	
2	47	12,208.32	12,411.57	0.56
3	68	11,959.72	12,253.78	0.87
*4*	*90*	*11*,*848*.*87*	*12*,*238*.*06*	*0*.*85*
5	113	11,816.23	12,304.89	0.80

*AIC* Akaike information criterion, *BIC* Bayesian information criterion. The optimal latent class model is highlighted in italic

The four behavioral classes generated were class 1 (*The substance and drug abstinent low risk takers*), class 2 (*The physically very active and moderate risk takers*), class 3 (*The high risk takers with poor hygiene*), and class 4 (*The highest risk takers*). Below is a description of the composition and characteristics of these four behavioral classes.

#### Class 1 (the Substance and Drug Abstinent Low Risk Takers)

This behavioral class comprised the majority (52%) of the adolescent study participants (see Table 2). These adolescents reported the lowest occurrence of alcohol, tobacco, and other drug use behavior (0–4.8%). They also reported the least occurrence of gambling behavior. The adolescents in class 1 reported the least engagement in sexual risk behavior, although their reports were similar to those from the adolescents in class 2. The reports of injury and violence-related behavior in this first class were generally lower than in classes 3 and 4, but comparable to the reports in behavioral class 2, with exception of engagement in physical fights and being bullied (reports on bullying were lower in class 1 compared to 2) and dating violence (reports on dating violence were lower in class 2 compared to 1). However, the adolescents in behavioral class 1 reported low levels of engagement in moderate and vigorous physical activity (i.e., less than 2 days per week of at least 10 min of physical activity). Their level of engagement in physical activity was significantly lower than that reported in the behavioral classes 2 and 4. Their reports of hygiene behavior were comparable to those reported in behavioral class 2, but much better than those of class 3.

**Table 2:** A comparison of behavioral characteristics across the four behavioral clusters

Variable	Class 1 (the substance and drug abstinent low risk takers*, n =* 291) *N* (%)	Class 2 (the physically very active and moderate risk takers, *n =* 135) *N* (%)	Class 3 (the high risk takers with poor hygiene, *n = 99*) *N* (%)	Class 4 (the highest risk takers, *n = 33*) *N* (%)	*P* value
Injury and violence-related behavior					
Had a serious injury during past 12 months (*Once or more than once)*	96 (32.9)^n,i^	48 (35.6)^q,m^	33 (33.3)^l^	21 (63.6)^k^	0.006
Engaged in a physical fight in past 12 months (*Once or more than once)*	79 (27.2)^h,c^	55 (40.7)^q,g^	38 (38.4)^l^	22 (66.7)^k^	< 0.001
Have ever been victims of forced sexual intercourse	6 (2.06)^b,c^	3 (2.2)^d,m^	19 (19.2)^s^	7 (21.2)^e^	< 0.001
Were victims of dating violence (*physical and*/*or sexual*) in past 12 months	4 (1.4)^b,c^	0 (0.0)^d,m^	11 (11.1)^l^	13 (39.4)^e^	< 0.001
Were victims of bullying in past 12 months	50 (17.2)^b,c^	47 (34.8)^d,a^	59 (59.6)^s^	18 (54.5)^k^	< 0.001
Had suicidal behavior (*suicide ideation and/or attempted suicide*) in the past 12 months	0 (0.0)^b,c^	0 (0.0)^d^	13 (13.1)^s^	8 (24.2)^e^	< 0.001
Alcohol, tobacco, and drug use behavior					
Have ever used tobacco and/or alcohol	4 (1.4)^n,c^	1 (0.7)^q,m^	3 (3.0)^f^	14 (42.4)^e^	< 0.001
Recently (*past 30 days*) used tobacco and/or alcohol	0 (0.0)^b,c^	4 (2.9)^j,g^	10 (10.1)^l^	11 (33.3)^k^	< 0.001
Have ever used marijuana and/or khat	0 (0.0)^b,c^	0 (0.0)^d^	11 (11.1)^f^	19 (57.6)^k^	< 0.001
Recently (*past 30 days*) used marijuana and/or khat	0 (0.0)^b,c^	0 (0.0)^j^	7 (7.1)^f^	17 (51.5)^e^	< 0.001
Have ever used other drugs	14 (4.8)^b,o^	15 (11.1)^d,g^	58 (58.6)^f^	4 (12.1)^r^	< 0.001
Sexual risk behavior					
Engaged in early sexual debut (*before 14 years*)	4 (1.4)^h,i^	0 (0.0)^j,m^	8 (8.1)^l^	11 (33.3)^e^	< 0.001
Engaged in transactional sex in past 12 months	3 (1.0)^h,o^	2 (1.5)^j,m^	12 (12.1)^s^	2 (6.1)^r^	< 0.001
Did not use a condom during their most recent sex intercourse	0 (0.0)^b,c^	0 (0.0)^d^	11 (11.1)^s^	8 (24.2)^e^	< 0.001
Dietary behavior					
Rarely or never eat fruits and vegetables during a regular week	38 (13.06)^b,o^	11 (8.2)^d,m^	31 (31.3)^l^	1 (3.0)^r^	< 0.001
Usually (*5*–*7 days per week*) consume fatty or fast foods during a regular week	32 (11.0)^h,c^	44 (32.6)^j,a^	18 (18.2)^s^	11 (33.3)^r^	< 0.001
Physical activity					
Days per week of at least 10 min of vigorous physical activity	1.5(±1.7)^c,n^	3.9 (± 2.7)^d,a^	1.8 (± 2.1)^f^	4.8 (± 2.4)^r^	< 0.001
Days per week of at least 10 min of moderate physical activity	1.4(±1.2)^h,c^	6.1 (± 1.2)^d,a^	0.9 (± 1.1)^f^	4.5 (± 1.8)^e^	< 0.001
Spend 5 h or more on sedentary activities during a typical day	29 (9.9)^n,i^	48 (35.6)^d,a^	8 (8.8)^l^	8 (24.2)^r^	< 0.001
Gambling behavior					
Ever spent much more than they planned on gambling activities within the past 12 months	7 (2.4)^b,c^	15 (11.1)^j,a^	22 (22.2)^s^	8 (24.2)^k^	< 0.001
Hygiene behavior					
Inadequate oral hygiene (*never cleans/brush teeth or does on only some days in a regular week*)	51 (17.5)^b,o^	22 (16.3)^d,m^	70 (70.7)^f^	3 (9.1)^r^	< 0.001
Inadequate general body hygiene (*never or rarely or only sometimes takes a bath with soap and water in a regular week*)	19 (6.5)^h,o^	7 (5.2)^j,m^	19 (19.9)^s^	5 (15.2)^r^	0.001

Other drugs included: cocaine, heroin, methamphetamines, steroid pills/shots without doctor’s prescription, and injection of any illegal drug into the body

±: standard deviation

^a^*p* < 0.001 for cluster 1 vs. 2

^b^*p* < 0.001 for cluster 1 vs. 3

^c^*p* < 0.001 for cluster 1 vs. 4

^d^*p* < 0.001 for cluster 2 vs. 3

^e^*p* < 0.001 for cluster 2 vs. 4

^f^*p* < 0.001 for cluster 3 vs. 4

^g^*p* ≥ 0.001 and *p* ≤ 0.05 for cluster 1 vs. 2

^h^*p* ≥ 0.001 and *p* ≤ 0.05 for cluster 1 vs. 3

^i^*p* ≥ 0.001 and *p* ≤ 0.05 for cluster 1 vs. 4

^j^*p* ≥ 0.001 and *p* ≤ 0.05 for cluster 2 vs. 3

^k^*p* ≥ 0.001 and *p* ≤ 0.05 for cluster 2 vs. 4

^l^*p* ≥ 0.001 and *p* ≤ 0.05 for cluster 3 vs. 4

^m^*p* > 0.05 for cluster 1 vs. 2

^n^*p* > 0.05 for cluster 1 vs. 3

^o^*p* > 0.05 for cluster 1 vs. 4

^q^*p* > 0.05 for cluster 2 vs. 3

^r^*p* > 0.05 for cluster 2 vs. 4

^s^*p* > 0.05 for cluster 3 vs. 4

#### Class 2 (the Physically Very Active and Moderate Risk Takers)

This behavioral class comprised 24% of the adolescent sample. Their reports on engagement in sexual risk behavior, injury, and violence-related behavior and hygiene behavior were much better than reports from classes 3 and 4, and minimally different from the reports from class 1 (see Table 2). However, this second class compared to behavioral class 1 had significantly poorer reports that indicated more frequent occurrence of physical fights (*p* = 0.005), experience of bullying (*p* < 0.001), lifetime use of other drugs (*p* = 0.02), recent tobacco and/or alcohol use (*p* = 0.01), fatty food intake (*p* < 0.001), and gambling behavior (*p* < 0.001). Noteworthy, adolescents from behavioral class 2 reported the highest engagement in moderate physical activity (on average 6 days of at least 10 min of activity per week) and their engagement in vigorous physical activity was significantly higher than that of adolescents in classes 1 and 3.

#### Class 3 (the High Risk Takers with Poor Hygiene)

This behavioral class comprised 18% of the adolescents. Overall, they reported greater occurrence of violence and injury-related behaviors (11.1–59.6%), substance use (3.0–58.6%), sexual risk behavior (8.1–12.1%), gambling behavior (22.2%), poor fruit and vegetable consumption (31.3%), and inadequate hygiene behavior (19.9–70.7%) compared to members of behavioral classes 1 and 2 (see Table 2). Strikingly, reports from the majority (71%) of the adolescents in class 3 indicated inadequate oral hygiene and 59% reported lifetime use of other drugs. The occurrence of both of these health risk behaviors was significantly higher than in any other behavioral class. The adolescents of class 3 also reported the poorest levels of engagement in moderate physical activity, and they showed a similar level of engagement in vigorous physical activity as reported in class 1.

#### Class 4 (the Highest Risk Takers)

This behavioral class comprised the smallest proportion (6%) of the adolescent study sample. The adolescents in this class presented with significantly higher reports of injury and violence related behavior, sexual risk behavior, alcohol, tobacco and other drug use, and gambling behavior as compared to reports from classes 1 and 2. Although there was some extent of similarity in the burden of risky behavior between class 4 and 3, higher reports of serious injuries in the past 12 months (*p* = 0.002), fights (*p* = 0.005), dating violence (*p* = 0.001), early sexual debut (*p* < 0.001), both recent and lifetime use of tobacco/alcohol and marijuana/khat (*p* < 0.001–*p* = 0.004), and sedentary hours (*p* = 0.014) were found in class 4 compared to class 3. However, the adolescents in class 4 reported the lowest rates of inadequate oral hygiene (9.1%) and they did have the lowest reports on poor fruit and vegetable consumption (3%) across the four behavior classes. They also reported significantly higher levels of engagement in moderate and vigorous physical activity compared to the adolescents in class 1 and 3 (see Table 2).

### Socio-demographic, Biological and Psycho-Social Characteristics of the Four Behavioral Classes

Results from bivariate analysis of various factors across the behavioral classes are summarized in Table 3. No significant differences were found in HIV group distribution across the four classes (*p* = 0.69). About a quarter of the adolescents in each HIV group (26% of perinatally HIV infected, 20% of perinatally HIV exposed but uninfected, and 25% of the HIV unexposed and uninfected) belonged to the two highest risk classes (i.e., classes 3 and 4). The majority of the adolescents in behavioral class 4 (the highest risk takers) were male and generally older (*p* = 0.004) than the adolescents in other behavioral classes. Across all four behavioral classes, class 3 (the high risk takers with poor hygiene) comprised the greatest proportion of young adolescents with the lowest educational achievement (75% below upper primary school) and the group with the highest rate of nutritional stunting (all differences were statistically significant). In class 1 (the substance and drug abstinent low risk takers), significantly fewer reports of caretaker tobacco use (15.1%) and adolescents’ mental distress in the past 12 months (11.7%) were found compared to the other three behavioral classes. School attachment was significantly better in class 2 (the high physically active moderate risk takers) compared to class 1 (*p* = 0.008) and social economic status was higher for the adolescents in class 2 compared to those in class 3 (*p* = 0.026). Adolescents in both class 1 and class 2 had significantly fewer reports of food insecurity and feeling unsafe in the neighborhood in comparison to the riskier behavioral classes of 3 and 4.

**Table 3:** Socio-demographic, psychosocial, and biomedical characteristics of participants in the 4 behavioral clusters

Factors	Class 1 (the substance and drug abstinent low risk takers, *n* = 291) *N* (%)	Class 2 (the physically very active and moderate risk takers, *n* = 135) *N* (%)	Class 3 (the high risk takers with poor hygiene, *n* = 99) *N* (%)	Class 4 (the highest risk takers, *n = 33*) *N* (%)	*P* value
HIV status	P^n,o^	P^q,m^	P^s^	P^r^	0.686
HIV uninfected unexposed	103 (35.4)	47 (34.8)	38 (38.4)	11 (33.3)	
Perinatally HIV exposed uninfected	90 (30.9)	37 (27.4)	21 (21.2)	10 (30.3)	
Perinatally HIV infected	98 (33.7)	51 (37.8)	40 (40.4)	12 (36.4)	
Adolescence stage	P^h,o^	P^j,m^	P^l^	P^r^	0.027
Early adolescence (10–14 years)	195 (67.1)	92 (68.1)	79 (79.8)	18 (54.5)	
Mid-adolescence (15–17 years)	96 (32.9)	43 (31.9)	20 (20.2)	15 (45.5)	
Sex (*males*)	126 (43.3)^n,i^	67 (49.6)^q,m^	46 (46.5)^l^	24 (72.7)^k^	0.013
Age (*years*)	13.7 (± 1.5)^n,i^	13.8 (± 1.5)^q,m^	13.4 (± 1.6)^l^	14.5 (± 1.5)^r^	0.004
Education	P^b,o^	P^d,m^	P^l^	P^r^	0.001
Not attending school	2 (0.7)	0 (0.0)	2 (2.0)	1 (3.0)	
Special school	2 (0.7)	0 (0.0)	1 (1.0)	0 (0.0)	
Lower primary school (pre-primary to class 5)	139 (47.7)	59 (43.7)	71 (71.7)	11 (33.4)	
Upper primary school (class 6–8)	134 (46.1)	62 (45.9)	23 (23.3)	17 (51.5)	
Secondary school	14 (4.8)	14 (10.4)	2 (2.0)	4 (12.1)	
Orphanhood	P^n,o^	P^q,m^	P^s^	P^r^	0.492
Both parents alive	191 (65.6)	98 (72.6)	65 (65.7)	22 (66.7)	
Only mother alive	52 (17.9)	24 (17.8)	20 (20.2)	5 (15.2)	
Only father alive	28 (9.6)	4 (2.9)	5 (5.0)	3 (9.1)	
Both parents died	20 (6.9)	9 (6.7)	9 (9.1)	3 (9.1)	
Caretaker uses substances (*tobacco use*)	44 (15.1)^h,i^	37 (27.4)^q,g^	28 (28.3)^s^	12 (36.4)^r^	0.003
Caretaker-adolescent positive interaction (*total score of tool ranges from 0 to 72*)	46.9 (± 7.7)^n,o^	47.2 (± 8.6)^q,m^	45.7 (± 8.7)^s^	44.3 (± 7.3)^r^	0.180
School attachment (*total score of tool ranges from 0 to 16*)	12.4 (± 2.3)^n,o^	13.2 (± 2.1)^q,g^	12.5 (± 2.7)^s^	12.5 (± 2.1)^r^	0.013
Peer to peer positive relationship (*total score of tool ranges from 0 to 12)*	9.1 (± 2.3)^n,o^	9.3 (± 2.3)^q,m^	8.8 (± 2.8)^s^	8.9 (± 2.3)^r^	0.359
Social economic status (*total score of tool ranges from 0 to 9)*	1.6 (± 1.4)^n,o^	2.0 (± 1.7)^j,m^	1.4 (± 1.3)^s^	2.2 (± 1.9)^r^	0.006
Food insecurity in past 30 days (*always*/*most of the time/sometimes going hungry due to no food at home*)	32 (11.0)^h,i^	23 (17.0)^q,m^	20 (20.2)^s^	8 (24.2)^r^	0.037
Feeling unsafe in their neighborhood	47 (16.2)^b,c^	24 (17.8)^j,m^	36 (36.4)^s^	15 (45.5)^k^	< 0.001
Mental distress in past 12 months (*feeling sad or hopeless almost every day for two weeks or more in a row*)	34 (11.7)^b,c^	28 (20.7)^j,g^	39 (39.4)^s^	13 (39.4)^k^	< 0.001
History of any other medical conditions (*Epilepsy*, *Cerebral Palsy*, *history of an acute illness*, *Sickle cell disease*, *Stroke*, *any hospital admissions*)	85 (29.2)^n,o^	40 (29.6)^q,m^	36 (36.4)^s^	13 (39.4)^r^	0.222
Being stunted	78 (26.8)^b,i^	31 (22.9)^d,m^	46 (46.5)^l^	7 (21.2)^r^	< 0.001
BMI for age	P^n,o^	P^q,m^	P^s^	P^r^	0.847
Thinness	52 (17.9)	22 (16.3)	20 (20.2)	3 (9.1)	
Overweight or obese	15 (5.2)	5 (3.7)	4 (4.0)	1 (3.0)	
CD4 Cell count (*specific for perinatally HIV infected n = 190*)	P^n,o^	P^q,m^	P^s^	P^r^	0.126
> 500 cells/mm^3^	56 (60.9)	39 (79.6)	25 (65.8)	1 (63.6)	
350–500 cells/mm^3^	11 (11.9)	4 (8.2)	7 (18.4)	3 (27.3)	
< 350 cells/mm^3^	25 (27.2)	6 (12.2)	6 (15.8)	1 (9.1)	
HIV viral load concertation (*specific for perinatally HIV infected n = 163*)	P^n,o^	P^q,m^	P^s^	P^r^	0.643
≤ 1000 copies/ml	38 (48.1)	14 (35.9)	16 (45.7)	4 (40.0)	

BMI: Body Mass Index

±: standard deviation

^a^*p* < 0.001 for cluster 1 vs. 2

^b^*p* < 0.001 for cluster 1 vs. 3

^c^*p* < 0.001 for cluster 1 vs. 4

^d^*p* < 0.001 for cluster 2 vs. 3

^e^*p* < 0.001 for cluster 2 vs. 4

^f^*p* < 0.001 for cluster 3 vs. 4

^g^*p* ≥ 0.001 and *p* ≤ 0.05 for cluster 1 vs. 2

^h^*p* ≥ 0.001 and *p* ≤ 0.05 for cluster 1 vs. 3

^i^*p* ≥ 0.001 and *p* ≤ 0.05 for cluster 1 vs. 4

^j^*p* ≥ 0.001 and *p* ≤ 0.05 for cluster 2 vs. 3

^k^*p* ≥ 0.001 and *p* ≤ 0.05 for cluster 2 vs. 4

^l^*p* ≥ 0.001 and *p* ≤ 0.05 for cluster 3 vs. 4

^m^*p* > 0.05 for cluster 1 vs. 2

^n^*p* > 0.05 for cluster 1 vs. 3

^o^*p* > 0.05 for cluster 1 vs. 4

^q^*p* > 0.05 for cluster 2 vs. 3

^r^*p* > 0.05 for cluster 2 vs. 4

^s^*p* > 0.05 for cluster 3 vs. 4

### EF Outcomes Across the Four Behavioral Classes

Results from the ANOVA to identify differences in EF outcomes across classes revealed that significant differences in performance on all the four EF tasks exist across the behavioral classes (see Table 4). The adolescents in class 3 had the lowest scores (*p* < 0.001) on both tasks of working memory (i.e., Backward digit span and LNS). The adolescents in class 3 also had the lowest mean T-scores on cognitive flexibility and they had the highest Stroop interference scores, but these differences were only statistically significant in comparison to scores for the lower risk classes (i.e., classes 1 and 2).

**Table 4:** Summary of scores of executive functioning (backward digit span, letter-number sequencing, Stroop interference, and comprehensive trail making) and their association with behavioral cluster membership

Executive functioning	Class 1 (the substance and drug abstinent low risk takers, *n =* 291) *N* (%)	Class 2 (the physically very active and moderate risk takers, *n* = 135) *N* (%)	Class 3 (the high risk takers with poor hygiene, *n* = 99) *N* (%)	Class 4 (the highest risk takers, *n* = 33) *N* (%)	*P* value^a^
Backward digit span (*total correct raw scores*)^b^	3.7 (± 2.9)	4.2 (± 2.8)	2.2 (± 2.4)	4.3 (± 3.3)	< 0.001
Letter-number sequencing (*total correct raw scores*)^c^	4.9 (±2.3)	5.2 (± 2.3)	3.3 (± 1.4)	4.8 (± 2.3)	< 0.001
Stroop Interference (*raw scores*)^d^	− 16.1 (± 11.0)	− 16.9 (± 9.9)	− 12.8 (± 10.8)	− 15.7 (± 9.6)	0.025
Comprehensive trail making (*mean T-scores*)^e^	24.9 (± 5.1)	25.5 (± 5.1)	22.9 (± 3.9)	24.8 (± 5.6)	0.0007

±: Confidence Interval

^a^*P* values for the post-hoc analysis (Bonferroni correction) in ANOVA

^b^Backward digit span- measures working memory (increasing scores reveal better working memory)

^c^Letter-number sequencing—measures working memory (increasing scores reveal better working memory)

^d^Stroop interference—measures inhibitory control (increasing scores reveal worse inhibitory control abilities)

^e^Comprehensive trail making—measures cognitive flexibility (increasing scores reveal worse cognitive flexibility)

### Findings from DAG

The minimum sufficient adjustment sets, which were the identified variables for adjustment, from the DAG (see Fig. 1) were executive functioning, experience of mental distress, household socio-economic status, parent-to-adolescent interaction, feeling unsafe in their neighborhood, age, and current educational level. These variables were adjusted in the model for estimating the effect of perinatal HIV status on HRB clustering.

### Association Between Perinatal HIV Status and Behavioral Class Membership

The factors associated with behavioral class membership are summarized in Table 5. The results from an adjusted multinomial logistic regression model indicate that working memory (as measured by the letter number sequencing task) (*p* = 0.0037), adolescent’s experience of mental distress within the past 12 months (*p* < 0.001), adolescent’s feeling of insecurity within the neighborhood (*p* = 0.002), and age of the adolescent (*p* = 0.047) are significantly associated with HRB clustering. Noteworthy, the risk of belonging to behavioral class 3 relative to class 1 reduces by 27% for every unit increase in a score of working memory as measured on the letter number sequencing task (RRR = 0.73, *p* < 0.001). The risk of belonging to classes of higher behavioral risk (i.e., classes 2, 3, and 4) relative to class 1 increases twice or higher among adolescents who experienced mental distress within the past 12 months and the results are statistically significant across all the behavioral classes. The risk of belonging to the behavioral classes 3 and 4 relative to class 1 increases more than twice among adolescents who feel unsafe in their neighborhood. A unit increase in age of the adolescent was associated with 38% increase in risk of belonging to behavioral class 4 relative to class 1 (RRR = 1.38, *p* = 0.037). Finally, although perinatal HIV status did not have an overall significant association with HRB cluster membership (*p* = 0.366), there was borderline significance (*p* = 0.045) for a protective effect against membership to class 3 relative to class 1 for adolescents who were perinatally HIV exposed but uninfected (RRR = 0.49).

**Table 5:** Results from a multinomial logistic regression model showing the association between adolescents’ perinatal HIV status and membership to behavioral clusters

	RRR, 95% confidence interval	*P* value	RRR, 95% confidence interval	*P* value	RRR, 95% confidence interval	*P* value	Overall *P* value
Variable^a^	Class 2 vs. class 1		Class 3 vs. class 1		Class 4 vs. class 1		
HIV status^b^							0.366
Perinatally HIV exposed uninfected	0.8 (0.46, 1.38)	0.42	0.49 (0.25, 0.98)	0.045	1.37 (0.50, 3.72)	0.54	
Perinatally HIV infected	1.1 (0.66, 1.85)	0.70	0.66 (0.36, 1.23)	0.195	1.00 (0.38, 2.67)	0.99	
Letter-number sequencing (*total correct raw scores*)	1.0 (0.89, 1.13)	0.90	0.73 (0.61, 0.87)	*p* < 0.001	0.89 (0.71, 1,12)	0.32	0.0037
Mental distress in past 12 months (*No distress*—*reference*)	1.9 (1.12, 3.49)	0.019	3.86 (2.15, 6.93)	*p* < 0.001	3.82 (1.64, 8.91)	0.002	< 0.001
Feeling unsafe in their neighborhood (*Feeling safe*—*reference*)	1.09 (0.61, 1.91)	0.77	2.09 (1.18, 3.73)	0.011	3.87 (1.69, 8.8)	0.001	0.002
Age (*years*)	0.97 (0.82, 1.17)	0.82	0.86 (0.69, 1.05)	0.15	1.38 (1.02, 1.88)	0.037	0.047

*vs* versus, *RRR* relative risk ratio

Class 1: the substance and drug abstinent low risk takers; class 2: the physically very active and moderate risk takers; class 3: the high risk takers with poor hygiene; class 4: the highest risk takers

^a^Only variables that were statistically significant or had a statistically significant category are presented

^b^HIV uninfected unexposed is the reference group for HIV status

## Discussion

In the present study, we identified four distinct behavioral classes with varying levels of HRB burden and clustering among an adolescent sub-population from a low resource setting in Kenya. The majority (slightly more than a half) of these adolescents belonged to the class with the least reports of HRB occurrence and clustering. However, we found that almost a quarter of the adolescents belonged to two behavioral classes with the highest occurrence of multiple forms of HRB. This segment of adolescents may potentially represent a vulnerable sub-population with shared cumulative psychosocial risk factors which underlie an unhealthy lifestyle [10]. Our findings of the four class solution are similar to reports from other studies on HRB clustering conducted among rural Chinese [79], Canadian [80], and Dutch adolescents [11]. The proportion of adolescents in the highest risk class (5.9%) in this present study was close to composition of the highest risk group (4%) among rural Chinese adolescents [79], whose mean age (14.7 years) was also similar to the age of our participants (13.7 years). However, these findings should be interpreted cautiously since the other studies did not focus on adolescents living with HIV, were from different geographical settings, and measured behaviors that were different from the ones reported in this study. This stated, our findings do emphasize that clustering of HRB among adolescents in rural coastal Kenya is of concern. More importantly, there exists a segment of the adolescents with heightened vulnerability to multiple forms of HRB and thus targeted risk reduction and prevention interventions are urgently required to address this problem.

Another key finding is that our hypothesis that perinatal HIV infection is associated with HRB clustering was not supported by the study findings. Overall, we did not find a significant association between perinatal HIV status (regardless of the HIV specific biomarkers) and HRB clustering. We had suggested that HIV infection introduces extra adversity and HIV associated neurocognitive deficits, which could heighten vulnerability for HRB. We propose two plausible explanations for the absence of this association. First, the access to services such as proper counseling, socio-emotional support, and home visitation received through HIV care and treatment clinics potentially lessens the impacts of psychosocial adversity among adolescents living with HIV, thereby enhancing their healthy lifestyle [81]. Noteworthy, all the adolescents living with HIV in our study were enrolled in comprehensive HIV care and treatment clinics. Second, we suggest that adolescents who experienced HIV-associated neurocognitive impairment may benefit from HIV medication and experience-dependent neuroplasticity across the lifespan, which improves their resilience and lessens the severity of long-term consequences (e.g., EF deficits) and the implications on their mental and behavioral outcomes [82]. Notably, our findings on the lack of differences in HRB outcomes across the HIV groups in our study are consistent with previous studies from SSA [3] and the USA [19]. These findings stress that children born to HIV-infected women are equally at risk for multiple forms of HRB, which emphasizes the need for adolescent-friendliness of public health services. Such services would help to attract young people regardless of their HIV status and to retain them in continuous care. Guidelines for provision of adolescent youth friendly services in Kenya were formulated in 2005 [73]; however, more effort is still needed to improve coverage of adolescent friendly services within the Kenyan healthcare system [83].

Although we found various significant differences in behavior composition across the four classes, there was generally a high and cross-cutting burden of various forms of injury and violence related behavior, poor hygiene, inadequate physical activity, and poor dietary behavior. Similar to these findings, poor sanitation and hygiene conditions [84], dietary and physical activity transition [85], and a high burden of violence-related behavior, such as bullying [86], have been reported among adolescents in Kenya in other studies. It is plausible that extreme household poverty coupled with psychosocial adversity are highly prevalent in this study setting and potentially predispose the general adolescent sub-population to shared forms of HRB. Indeed, some indication of shared vulnerability and adversity in this setting is illustrated by our findings of extremely low mean scores (1.8 out of the maximum 9) on the socio-economic status index, coupled by the high proportion (32%) of orphaned adolescents (without differences by HIV status), frequent reports of food insecurity (18%), and low levels of caretaker-adolescent positive interaction (an average score of 64% on the scale we utilized in the study). All these factors combined are likely to exacerbate mental distress among the adolescents, and a large body of evidence has linked mental distress to HRB in adolescence [25, 26]. Corroboratively, we found that the risk of membership to behavioral classes with a greater burden of HRB clustering increased when adolescents reported a history of mental distress within the past 12 months. Noting that most of the factors which could increase psychosocial adversity are household or family level factors, there is a need for appropriate interventions that enhance good parenting practices such as parental or caregiver involvement and responsive communication and which incorporate aspects on poverty alleviation.

Another key finding is that our assumption that deficits in EF may predispose adolescents to HRB clustering was supported by our study findings. However, this effect was not HIV group specific as we presupposed. Specifically, we found that better working memory and cognitive flexibility were associated with a lower burden of HRB clustering. Moreover, the protective effects of working memory still persisted after multivariate analysis. Similar to the findings in this present study, stronger working memory has been shown to regulate adolescents’ HRB, like substance use [35], and sexual risk taking [36]. Plausibly, the effects of cognitive flexibility could be masked by the effects of educational achievement because these two are closely linked [87]. These findings stress the need to incorporate interventions which stimulate EF development in preventive/behavioral interventions. Of note, such interventions have already been found beneficial when they also address social, physical, and emotional needs [88]. Hence, our findings add to the existing literature regarding the link between EF and HRB [35, 89].

The findings from our study indicate that a high burden of victimization, internalizing problems, and suicidal behavior distinguished the adolescents within the top two risky behavioral classes. Victimization (sexual, physical, emotional) [90], emotional, and behavioral problems [91] are commonly reported as major concerns for adolescents within the Kenyan setting. The findings on clustering of victimization, suicidal behavior, and other forms of HRB like sexual risk and substance use among the adolescents in behavioral classes 3 and 4 are corroborated by an existing body of research showing that victimized adolescents may experience an elevated burden of suicidal behavior [92] alongside maladaptive behavior such as substance use [93]. Nonetheless, more research is needed to better understand the pathways and specific associations between victimization and engagement in forms of HRB. Additionally, screening for victimization during adolescence and the provision of post-violence and mental health services should be incorporated among routine adolescent health promotion programs within this study setting.

Finally, we found that older adolescents are at an increased risk of membership to a behavioral class of higher HRB burden. This finding is consistent with results from other studies on multiple HRB among adolescents [94, 95]. Hence the relevance of age-appropriate interventions when addressing HRB during adolescence is highlighted. This finding also suggests that it may not be sufficient to collate adolescent age (10–19 years) into a single set when studying health behavior and lifestyle. Rather, adolescence should be sub-categorized into different developmental stages like early and late adolescence to better understand health outcomes and specific needs of this sub-population.

### Strengths and Weaknesses

To the best of our knowledge, this is the first study from SSA that investigates adolescents’ HRB clustering, utilizing a variety of behaviors, which simultaneously involves adolescents from three HIV groups (i.e., perinatally HIV infected, perinatally HIV exposed but uninfected, and HIV unexposed and uninfected). The study also elaborates the role of EF in HRB clustering; an area that has not been previously studied within the adolescent sub-population in SSA. Nonetheless, this study was conducted among adolescents from a poor rural setting and of a young age (mean = 13.7 years), who may not necessarily be representative of the general adolescent sub-population in Kenya, for example those living in urban and less impoverished backgrounds. Also, the adolescents living with HIV were perinatally infected, were all enrolled in HIV care, and had been fully disclosed of their HIV status. It is plausible that their HRB outcomes and/or predictors differ from those of adolescents who acquired HIV behaviorally or those who are not enrolled in treatment or not aware of their HIV status. Nonetheless, aggregating perinatally HIV infected and behaviorally infected adolescent groups could potentially blur unique outcomes and this has been cautioned against [96]. Furthermore, this study so far was based on a cross-sectional design, which makes directionality of associations challenging to ascertain. HRB was self-reported and this could have introduced some social desirability bias. However, such bias was to a large extent countered by the ACASI method of questionnaire administration. This is because this method does not involve face-to-face interview delivery, but rather an audio-recorded voice guides the participant through the interview questions, which enhances privacy, especially when reporting on sensitive topics.

## Conclusion

Despite some limitations, the present findings have important implications for clinicians and policy makers. First, adolescents born to HIV-infected mothers from poor rural settings are equally as vulnerable to HRB as their uninfected peers. This highlights the urgent need for inclusive and multi-component interventions which are tailored to the adolescents’ early or later developmental stage, and simultaneously address multiple forms of HRB. Such interventions could be made more inclusive and comprehensive by implementing them within rural adolescent HIV primary health clinics. Caregivers and family members could be actively involved through program components that address household psychosocial risk factors like poverty; and by incorporating services to promote mental health and EF development. Future longitudinal studies are needed to examine mechanisms which underlie HRB clustering and the changes in lifestyle that occur across the adolescents’ developmental stage.
